# Chemical composition and larvicidal efficacy of essential oils from three artemisia species endemic to the Mediterranean region against *Culex pipiens* (L.), insecticide-resistant vector: *in vivo* and *in silico* studies

**DOI:** 10.3389/fpls.2026.1742643

**Published:** 2026-03-09

**Authors:** Khalid Chebbac, Fatimazahra Guerguer, Mohammed Chalkha, Abdelfattah El Moussaoui, Mohammed Bassouya, Soufyane Lafraxo, Na’il Saleh, Zineb Benziane Ouaritini, Samir Chtita, Raja Guemmouh

**Affiliations:** 1 Laboratory of Biotechnology and Preservation of Natural Resources, Faculty of Sciences Dhar El Mahraz, Sidi Mohammed Ben Abdallah University, Fez, Morocco; 2 Laboratory of Analytical and Molecular Chemistry, Faculty of Sciences Ben M’Sik, Hassan II, University of Casablanca, Casablanca, Morocco; 3 Laboratory of Materials Engineering for the Environment and Natural Resources, Faculty of Sciences and Techniques, University of Moulay Ismail of Meknès, Errachidia, Morocco; 4 Engineering Laboratory of Organometallic, Molecular Materials and Environment, Faculty of Sciences Dhar EL Mahraz, Sidi Mohamed Ben Abdellah University, Fez, Morocco; 5 Plant Biotechnology Team, Faculty of Sciences, Abdelmalek Essaadi University, Tetouan, Morocco; 6 Laboratory of Biotechnology, Environment Agrifood and Health, Faculty of Sciences Dhar El Mahraz, Sidi Mohamed Ben Abdellah University, Fez, Morocco; 7 Department of Chemistry, College of Science, United Arab Emirates University, Al Ain, United Arab Emirates; 8 Laboratory of Natural Substances, Pharmacology, Environment, Modeling, Health and Life Quality, Faculty of Sciences Dhar El Mahraz, Sidi Mohammed Ben Abdellah University, Fez, Morocco

**Keywords:** *Culex pipiens*, endemic *Artemisia* species, essential oil, *in vivo* larvicidal efficacy, molecular docking, molecular dynamics

## Abstract

**Objective:**

This study aims to evaluate the larvicidal efficacy of essential oils extracted from three North African endemic *Artemisia* (*A*) species against the *Culex pipiens* mosquito, a major vector of emerging or re-emerging viruses that pose a threat to public and veterinary health in Africa, using both *in vivo* and *in silico* approaches.

**Methods:**

Third- and fourth-instar larvae were exposed to varying concentrations of essential oils from the selected *Artemisia* species. Lethal concentrations LC_10_, LC_50_, and LC_90_ for each essential oil were determined through probit analysis. The susceptibility of *C. pipiens* was also compared to that of the standard insecticide, temephos, under controlled laboratory conditions. Additionally, *in silico* studies, including molecular docking and dynamics simulations, were conducted on the major chemical constituents to further interpret and explain the *in vivo* results.

**Results:**

Gas chromatography analyses revealed that the essential oil of *A. negrei* was dominated by β-thujone (29.02%) and camphor (14.68%). In *A. herba-alba* Asso, α-thujone (49.19%) was the predominant compound, followed by terpinen-4-ol (9.82%). The essential oil of *A. campestris* was mainly characterized by camphor (33.07%) and 1,8-cineole (5.26%). *A. negrei* exhibited the highest larvicidal activity against *C. pipiens*, followed by *A. campestris.* In contrast, the essential oil of *A. herba-alba* Asso, despite its richness in oxygenated monoterpenes, showed the lowest larvicidal effectiveness. *In silico* investigations revealed that the examined compounds had promising binding affinities within the receptor’s active site, comparable to those of the reference insecticide, temephos.

**Conclusions:**

The results of this study suggest that *A. negrei*, an endemic species from Morocco, holds significant potential for managing *C. pipiens* populations and provides a promising alternative to chemical insecticides.

## 1 Introduction

Vector-borne diseases, caused by bacterial, viral, or parasitic infections, account for more than 17% of global infectious diseases and result in over a million deaths each year (Zientara et al., 2020; Jansen et al., 2023). Mosquitoes, key vectors of diseases such as malaria, dengue, leishmaniasis, and numerous arboviruses, play a crucial role in transmission (Harbach, 2022). Among the 3500 mosquito species, only a few, often anthropophilic, are responsible for transmitting these viruses by exploiting larval development sites created by humans (Brugman et al., 2018).

*C. pipiens* is a mosquito species widely recognized as a vector of the West Nile virus (WNV). It is also one of the most widespread mosquitoes globally due to its adaptation to the human environment and its feeding habits, which reinforce its role in the transmission of the West Nile virus and other pathogens, in addition to causing local and systemic allergic reactions in humans (Cheng et al., 2008; Farajollahi et al., 2011; Zahran et al., 2017a).

In Morocco, *C. pipiens* is strongly suspected of being the primary vector for the transmission of the WNV, with reported outbreaks in 1996, 2003, and 2010 (Chebbac et al., 2024b). These outbreaks have raised significant public health concerns, as WNV can cause severe neurological diseases, including encephalitis (Krzyzowska et al., 2024). The species thrives in both rural and urban areas, where it breeds in human-made containers, which aids in the virus’s transmission cycle (Arich et al., 2021). Migratory birds, which can carry the virus, also contribute to the geographic spread of WNV in the region. Enhanced vector control and surveillance are necessary to mitigate the health risks posed by this mosquito species (Brugman et al., 2018).

The excessive use of synthetic insecticides, combined with a lack of effective pesticide use strategies, leads to mosquito resistance to chemical treatments. This resistance not only increases risks to human and animal health but also contributes to environmental pollution (Murgue et al., 2001; Gallian et al., 2005; Gubler, 2007). Given these challenges, it is crucial to find alternatives to chemical insecticides.

The genus *Artemisia* comprises several hundred species that have been traditionally used for their antirheumatic, antispasmodic, anti-inflammatory, antimicrobial, and antihelmintic properties (Chebbac et al., 2024a). Among them, certain species such as *A. campestris*, *A. herba-alba* Asso, and *A. negrei* are considered endemic to Morocco, according to the International Union for Conservation of Nature (IUCN) assessment for North Africa (Eybpoosh et al., 2019). In Morocco, species of the genus *Artemisia* are widely distributed across arid and semi-arid regions, particularly in the High Atlas, Middle Atlas, Anti-Atlas, and Saharan zones, where they form an essential component of steppe and desert vegetation (Bougoutaia et al., 2021).

Previous studies have shown that certain essential oils extracted from *Artemisia* species have insecticidal properties, including activity against mosquitoes that transmit diseases such as *Anopheles* and *Aedes*, which justifies investigating their effects on *C. pipiens* (Luo et al., 2022; Alami et al., 2025). However, limited research has been conducted on the efficacy of these oils against this particular species, especially within the African context. Therefore, this study aims to evaluate the insecticidal potential of essential oils from *A. negrei*, *A. campestris*, and *A. herba-alba* Asso, collected from Jbel Bounacer in the Middle Atlas of Morocco, against *C. pipiens*.

## 2 Materials and methods

### 2.1 Plant collection and essential oil extraction of the *Artemisia*

The plants studied were collected in 2019 in the Middle Atlas Mountains, in eastern Morocco (33.539648, −3.894474). Botanical identification was carried out, and reference specimens were deposited at the Department of Biology of the Faculty of Sciences under the following reference numbers: *A. negrei* (BPRN/04/18), *A. campestris* (BPRN/05/19) and *A. herba-alba* Asso (BPRN/06/19). After collection, the plant materials were air-dried in the shade for about seven days in a dry, well-ventilated area and protected from direct sunlight to prevent essential oil degradation caused by heat and light exposure. To extract the essential oils, 100 grams of dried leaves from each plant were finely ground into a powder. Hydrodistillation was then carried out using a Clevenger apparatus for 2 to 3 hours, with careful monitoring of the temperature and water levels during the process.

After hydrodistillation in a closed Clevenger apparatus, the essential oils were separated by density-based decantation, with the oil layer floating above the hydrolat. The oils were then dried with anhydrous sodium sulfate to remove any residual moisture. Finally, the essential oils were stored in small opaque glass bottles at a temperature of 4 ± 2 °C to preserve their chemical integrity and prevent oxidation (Elyemni et al., 2019).

### 2.2 Calculation of essential oil yield

The yield of essential oil was determined after the hydrodistillation of the plant leaves. After extraction, the essential oils were separated by decantation and weighed using an analytical balance. The yield was expressed as a percentage based on the mass of essential oil obtained relative to the initial mass of fresh plant material used. The following formula was applied to calculate the essential oil yield (Equation 1):

(1) Yield (%)=(Mass of essential oil/Mass of fresh plant material)×100

Where:

- Mass of essential oil: The final weight of the essential oil after distillation and removal of residual moisture.
- Mass of fresh plant material: The weight of the leaves before extraction.

This method is widely accepted for evaluating the efficiency of essential oil extraction techniques and is applicable to various plant materials (Rana et al., 2022).

### 2.3 Chemical study and identification of the compounds of EOs

The chemical composition of the essential oils was determined using gas chromatography coupled with mass spectrometry (GC–MS), enabling both chromatographic separation and qualitative identification of the volatile constituents. Analyses were carried out using a Shimadzu GCMS-TQ8040 NX Triple Quadrupole GC–MS system (Shimadzu, Tokyo, Japan) equipped with an apolar RTxi-5 Sil MS capillary column (30 m × 0.25 mm i.d., 0.25 µm film thickness). Prior to analysis, the essential oils were diluted in n-hexane (10:100, v/v). A volume of 1 µL of the diluted sample was injected in split mode (split ratio 1:20). Helium was used as the carrier gas at a constant flow rate of 1.0 mL min^−1^, and the injector temperature was maintained at 250 °C. The GC oven temperature program was set as follows: initial temperature at 50 °C for 2 min, followed by a ramp of 5 °C min^−1^ to 160 °C (held for 2 min), then increased at 5 °C min^−1^ to 280 °C (held for 2 min). The total analysis time was approximately 50 min. Mass spectrometric detection was performed in electron ionization (EI) mode at 70 eV. The ion source temperature was set to 200 °C, the interface temperature to 280 °C, and data were acquired in full-scan mode over the m/z range 40–650. To confirm the identities of the compounds, the observed Kovats retention indices were compared to the reference values from the NIST 98 database and the Adams database (Adams, 2017).

### 2.4 Larval habitat characteristics

*C. pipiens* larvae were collected from a breeding site located in the urban area of Fez (Center of Morocco), near a small tributary of the El-Gaada Dam (altitude: 407 m; coordinates: 34°01’155” N, 004°57’213” W) during the month of May. This site is characterized by a particularly high density of Culicidae larvae, including *C. pipiens*. It is located near an animal farm and a horse stable, which promotes the proliferation of *C. pipiens* larvae due to the availability of organic matter and favorable breeding conditions.

### 2.5 Collection of *C. pipiens* larvae

A plastic tray inclined at a 45° angle toward the water surface was used to collect the larvae. The surface tension generated by the tray attracts the larvae. The collected larvae were kept for reproduction in rectangular trays at an average temperature of 24.6 °C ± 4 °C and a relative humidity of 72% ± 5%, with a light/dark cycle of 14:10 hours, in the entomological laboratory of the University of Fes (Parker, 2020).

### 2.6 Identification of larvae

The key for identifying Culicidae in Morocco was used to determine the morphological characteristics of the larvae, including *C. pipiens* (Himmi et al., 1995). The software for identifying African Mediterranean arthropods was also used to determine the scientific names of the mosquito vectors to be tested (Rahola et al., 2022). The identification of the larvae was confirmed by entomologist Mrs. Raja Guemmouh.

### 2.7 Biocide tests

The biological tests were performed following the standard protocol proposed by the WHO (2005, 2016), with some modifications (World Health Organization, 2016, 2019). A 10% stock solution of each essential oil was prepared by dissolving the specific essential oil in ethanol, then diluting it with ethanol to obtain a range of concentrations: 25, 50, 75, and 100 mg/L for *A. negrei*; 100, 250, 500, 750, and 1000 mg/L for *A. campestris*; 250, 500, 750, 1000, and 1500 mg/L for *A. herba-alba* Asso. A positive control (temephos) was also prepared with a range of concentrations: 0.0006, 0.0012, 0.0025, 0.05, and 0.0625 mg/L. A total of 20 third- and fourth-instar larvae of *C. pipiens* were placed into plastic coffee cups (100 mL) filled with distilled water at the designated concentration. The larvae were not fed during the 24 h exposure period. Three repetitions were made for each concentration, and three controls were prepared for each test. The negative control consisted of 99 mL of distilled water and 1 mL of ethanol, with the same number of larvae. The beakers containing the larvae were kept in the laboratory under standard light conditions (photoperiod 14:10 h light/dark), at a temperature of 25 ± 3 °C and humidity of 67–75%. Mortality was recorded after 24 hours. LC_10_, LC_50_, and LC_90_ values were obtained using the Log-Probit software. Three repetitions were conducted for each dilution and for both controls. After 24 hours of exposure, the live and dead larvae were counted.

The concentration ranges tested for each *Artemisia* species were selected based on preliminary range-finding assays conducted prior to the definitive bioassays, as well as on differences in the intrinsic larvicidal potency of the essential oils. Initial screening tests were performed to determine the approximate concentration ranges causing partial mortality (between 10% and 90%) in *C. pipiens* larvae. Based on these preliminary observations, lower concentrations were selected for *A. negrei* due to its higher larvicidal activity, whereas higher concentration ranges were required for *A. campestris* and *A. herba-alba* Asso to obtain comparable mortality levels. This approach allowed the accurate estimation of LC_10_, LC_50_, and LC_90_ values for each essential oil while avoiding unnecessary exposure to ineffective or excessively lethal concentrations.

The results of the bioassay tests were expressed as the percentage of mortality according to the concentrations of the essential oils (biological insecticides) and the controls used. The mortality percentage for larvae exposed to the essential oils was corrected using Abbott's formula (Abbott, 1925). The corrected mortality was calculated as follows (Equation 2):

(2) $$\text{Corrected Mortality }\left(\%\right)=\frac{\%\text{Observed Mortality}-\%\text{ Control Mortality}}{100-\%\text{ Control Mortality}}\times 100$$

The test should be repeated if the control mortality exceeds 20%.

### 2.8 Data analysis

Data analysis was performed using the log-probit analysis software (Windl version 2.0), developed by CIRAD-CA/MABIS in October 1999 (Giner et al., 1999). This software automatically provides chi-square (χ²) values to assess the goodness-of-fit of the log-probit model to the observed larval mortality data. Additionally, ANOVA was used to analyze the variance, and mean values along with standard deviations were calculated for comparison. The chi-square values were used to verify the adequacy of the model, ensuring the reliability of the estimated LC50 and LC90 values.

### 2.9 *In silico* studies

#### 2.9.1 Molecular docking

The authors extracted essential oils from the *Artemisia* species studied and, in order to understand how their main compounds might act, conducted molecular docking studies to evaluate their potential efficacy, as well as that of temephos, against gamma-aminobutyric transaminase (GABA-T). The three-dimensional (3D) structure of GABA-T (PDB ID: 1SF2) was retrieved from the RCSB Protein Data Bank (Liu et al., 2004). Prior to the docking simulations, the protein structure was prepared by removing non-essential heteroatoms and adding polar hydrogen atoms for accurate hydrogen bonding. The 2D structures of the test compounds were subjected to geometric optimization using the MMFF94 force field and the steepest descent algorithm via Avogadro software. Docking calculations were performed using AutoDock Vina (Eberhardt et al., 2021), with the grid box centered on the active site of the protein, employing dimensions of 40 Å in each direction and a grid spacing of 0.375 Å. The specific coordinates used to define the active site were: X = 36.976133; Y = 35.121200; Z = 25.928000. Following the docking simulations, ligand–protein interactions were visualized and analyzed using BIOVIA Discovery Studio (Baroroh et al., 2023), highlighting specific interactions with key residues within the active site. Hydrophobic, ionic, hydrogen bonding, and Van der Waals interactions were taken into account in the calculation of binding free energy. A ligand was considered potentially active when the protein–ligand complex exhibited a significantly low interaction energy value (Agu et al., 2023).

#### 2.9.2 Molecular dynamics simulations

To assess the stability of the protein–ligand complexes and the structural fluctuations of the ligands, molecular dynamics simulations were performed on the selected compounds. These simulations were conducted using the Desmond v3.6 package in conjunction with the OPLS3e force field over a simulation period of 100 ns (Guerguer et al., 2025a). Desmond’s System Builder configured an orthorhombic water box with the TIP3P water model and a 10 Å buffer. To ensure electrostatic balance, Na+ and Cl− counterions were added. The simulation was performed at a constant temperature of 300 K and a pressure of 1 atm, with a thermostat relaxation time of 1 ns. Temperature and pressure were regulated using the Nose–Hoover chain thermostat and Martyna–Tobias–Klein barostat methods. Following the equilibration phase, a 100 ns simulation was run under the NPT ensemble. Stability parameters, including root mean square deviation (RMSD) and root mean square fluctuation (RMSF), were analyzed to assess protein stability and key interactions between ligands and essential residues.

## 3 Results and discussion

### 3.1 Yield of essential oils

The essential oil yields from the leaves of *A. negrei* (1.1%), *A. campestris* (0.75%), and *A. herba-alba* Asso (0.9%) species exhibit notable variations (**Table 1**), with *A. negrei* showing the highest yield among the three. In comparison to other *Artemisia* species, such as *A. frigida* (1.5%) and *A. cana* (1.3%), *A. negrei* has a relatively competitive yield, surpassing species like *A. absinthium* (0.3%-0.5%) (Zhang et al., 2014; Chebbac et al., 2021, 2024a). However, the yields of *A. campestris* and *A. herba-alba* Asso are in line with those of other less productive species in the genus, though they remain relevant for specific applications (Ekiert et al., 2022; Limam et al., 2024). When compared to industrial plants such as lavender (0.8%-2.8%) or rosemary (1%-2.5%), the yield of *A. negrei* is comparable and could offer promising commercial potential (Chebbac et al., 2021). While the yields of *A. campestris* and *A. herba-alba* Asso are relatively low, strategies such as optimizing harvest time, selecting high-yield genotypes, or improving cultivation and drying conditions could potentially increase their essential oil production. Nevertheless, their study is justified by the presence of specific bioactive compounds, which may provide added value for targeted applications. A detailed analysis of their chemical composition would further help evaluate their potential in medical or industrial fields (Hussain et al., 2024; Zhang et al., 2014).

**Table 1:** Extraction yields of essential oils from the leaves of three *Artemisia* species.

*Artemisia* species	Part used	Extraction method	Color/Odor	Yield (%)
*A. negrei*	Leaves	Hydrodistillation	Yellowish/Strong aroma	1.10 ± 0.05
*A. campestris*	Leaves	Hydrodistillation	Yellowish/Aromatic	0.75 ± 0.03
*A. herba-alba* Asso	Leaves	Hydrodistillation	Pale yellow/Intense aroma	0.90 ± 0.04

### 3.2 Chemical analysis of essential oils

GC/MS analyses of the essential oils from the three *Artemisia* species tested reveal distinct chemical compositions, which may influence their bioactive potential (**Table 2**).

**Table 2:** Phytochemical compounds identified in the essential oils of *A. negrei*, *A. herba-alba* Asso, and *A. campestris* by GC/MS analysis.

Retention index	Compound name	*A. negrei*		*A. campestris*		*A. herba-alba* Asso	
		RT (min)	Area (%)	RT (min)	Area (%)	RT (min)	Area (%)
939	*α*-Pinene	7.84	0.61	–	–	–	–
954	Camphene	8.22	2.38	8.368	2.48	25.970	5.24
979	β-Pinene	9.17	0.29	–	–	26.156	1.55
990	Myrcene	9.89	0.37	–	–	–	–
1026	p-Cymene	10.78	0.44	10.557	0.73	26.318	0.96
1029	Limonene	11.03	0.50	–	–	29.166	1.10
1031	1,8-Cineole	11.11	5.60	10.799	5.26	–	–
1083	Artemisia alcohol	13.16	0.50	–	–	14.080	1.70
1177	Terpinen-4-ol	–	–	15.244	0.90	25.922	9.82
1086	Fenchone	11.36	0.50	–	–	29.601	5.60
1102	α-Thujone	13.49	3.63	13.374	1.82	13.410	49.19
1114	β-Thujone	13.82	29.02	13.034	5.09	13.035	2.72
1121	p-Menth-2-en-1-ol	–	–	13.577	0.86	14.706	2.18
1133	Terpineol	16.54	0.35	–	–	18.281	8.27
1141	Verbenol	–	–	16.010	2.75	–	–
1146	Camphor	14.54	14.68	14.299	33.07	14.258	1.13
1164	Chrysanthenol	–	–	14.706	0.90		
1169	Borneol	15.65	3.85	14.999	3.47	14.997	1.0
1121	Isophorone	–	–	14.130	3.00	–	–
1238	Ocimenone	–	–	16.867	1.06	–	–
1252	Piperitone	–	–	17.327	0.73	–	–
1376	α-Copaene	26.15	1.00	–	–	–	–
1288	Bornyl acetate	19.85	0.51	–	–	14.020	0.79
1290	Cymen-7-ol	–	–	15.392	0.69	17.422	7.55
1298	Geranyl formate	20.81	0.88	–	–	15.798	1.22
1360	p-Mentha-8-thiol-3-one	–	–	17.750	2.83	–	–
1434	Coumarin	40.19	0.65			–	–
1479	γ-Muurolene	31.44	1.17	–	–		
1513	Cycloisolongifol-5-ol	29.43	2.88	–	–	–	–
1633	α-Acoreno	30.12	1.00	–	–	–	–
1641	Aromadendrene epoxide	28.70	1.20	–	–	–	–
1667	Limonen-4-ol	16.11	0.56	–	–	–	–
1718	Curcuphenol	37.17	1.09	–	–	–	–
1819	Trihydroxy benzaIdehyde	40.99	1.05	–	–	–	–
1829	Isopropyltetradecanoate	41.77	0.56	–	–	–	–
1845	Isotorquatone	41.91	1.43	–	–	–	–
1855	Lanceol acetate	42.56	0.95	–	–	–	–
1864	thujopsenic acid	42.69	1.54	–	–	–	–
1874	Hexadecanol	44.64	1.01	–	–	–	–
1960	Palmitic acid		1.72	–	–	–	–
2125	Octadecanoic acid, ethylester	44.56	0.60	–	–	–	–
2500	Pentacosane	43.57	3.07	–	–	–	–
2800	Octacosane	43.02	14.02	–	–	–	–

*A. negrei:* The essential oil presents a rich diversity of bioactive compounds, making it a promising candidate for insecticidal applications. Key components include α-thujone (3.63%) and β-thujone (29.02%), both known for their neurotoxic effects on insects (Nathan, 2007; Chebbac et al., 2021). Camphor (14.68%), octacosane (14.02%), and 1,8-cineole (5.6%) add to the potential mechanisms of action. Minor terpenes such as α-pinene (0.61%) and camphene (2.38%) contribute to insecticidal and repellent effects (Sanei-Dehkordi et al., 2016). while fenchone (0.50%) and limonene (0.50%) may enhance larvicidal activity (Cheng et al., 2003). Compounds like α-copaene (1.1%) and curcuphenol (1.01%) likely act synergistically, enhancing overall effectiveness against *C. pipiens*. The combination of α-pinene, camphor, and α-thujone appears to maximize larvicidal effects, providing significant potential for mosquito population management (Chebbac et al., 2024b). Overall, the predominance of oxygenated monoterpenes (61.03%) explains the high larvicidal activity observed.

*A. herba-alba* Asso: The essential oil is dominated by α-thujone (49.19%), with significant amounts of terpinene-4-ol (9.82%) and terpineol (8.27%), known for antimicrobial and insecticidal properties (Baker et al., 2023). Fenchone (5.60%), camphor (1.13%), and limonene (1.1%) complete its composition. While α-thujone is abundant, the absence of other key compounds found in *A. negrei* may limit larvicidal effectiveness. The high proportion of oxygenated monoterpenes (89.95%) likely explains its strong bioactivity, although its efficiency against *C. pipiens* appears lower than *A. negrei*, highlighting the importance of synergistic interactions between compounds such as α-pinene, camphor, and camphene (Baker et al., 2023).

*A. campestris*: This species has a high concentration of camphor (33.07%), with contributions from 1,8-cineole (5.26%) and β-thujone (5.09%). However, it lacks limonene and α-pinene, compounds associated with better larvicidal performance. Borneol (3.47%) and camphene (2.48%) may enhance activity, while menth-en-1-ol (2.83%) and isophoron (3%) suggest potential repellent effects. The dominance of oxygenated monoterpenes (62.40%) supports moderate larvicidal activity, but the absence of key synergistic compounds likely limits effectiveness compared to *A. negrei.*

These chemical profiles explain the differences in larvicidal activity among the three species. The presence of α-pinene, camphor, and α-thujone in *A. negrei* likely acts synergistically to maximize larvicidal potential. In *A. herba-alba* Asso, although α-thujone and limonene are abundant, the lack of α-pinene may reduce efficacy. *A. campestris* shows moderate activity due to missing synergistic compounds despite high camphor content. Overall, the results suggest that the composition and relative abundance of bioactive compounds are critical determinants of the essential oils’ larvicidal potential.

### 3.3 Larvicidal activity of the tested essential oils

**Figure 1:**
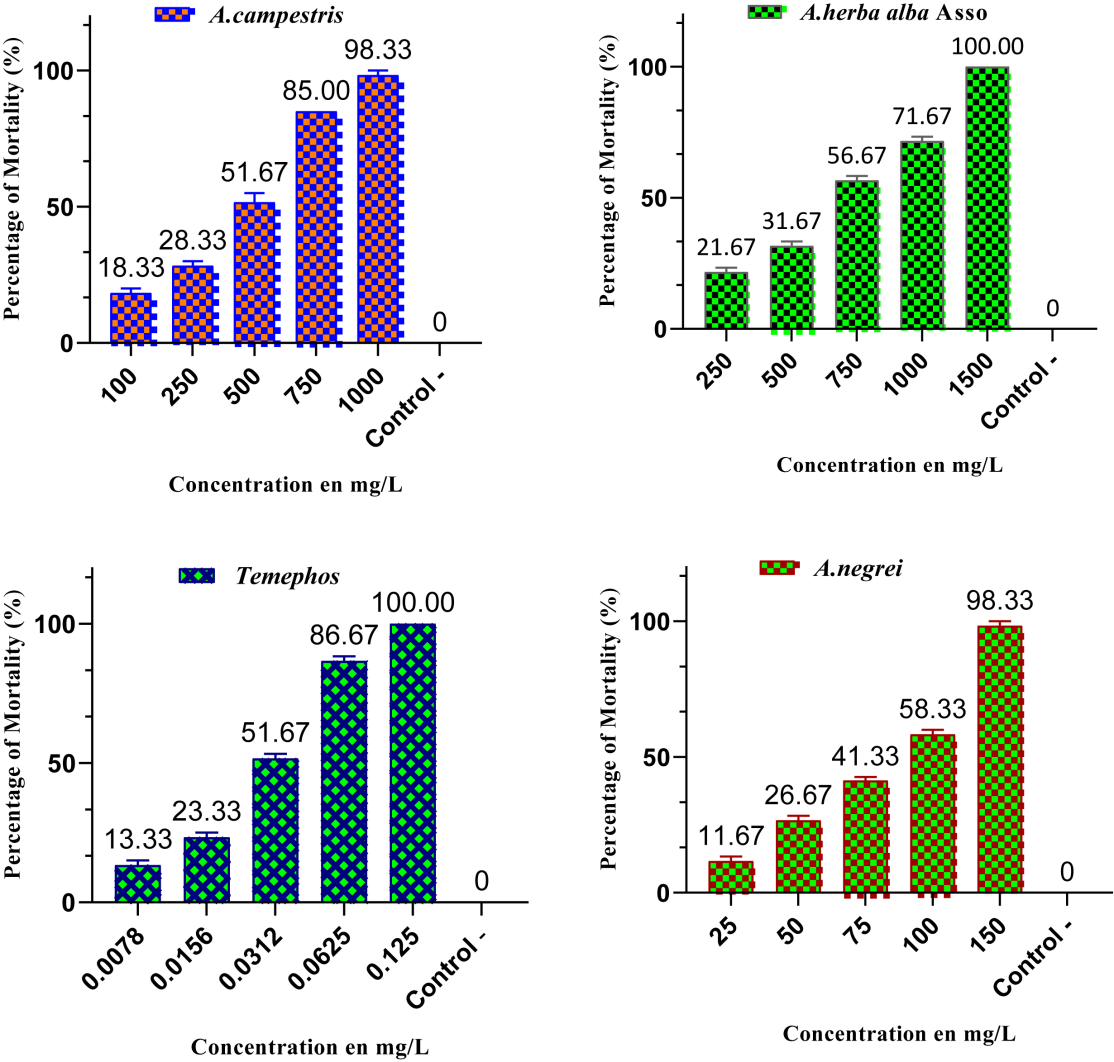
Mortality of *C. pipiens* larvae (%) induced by the essential oils of *A. negrei*, *A. herba-alba* Asso, and *A. campestris* at various concentrations after 24 hours of exposure (negative control: ethanol; positive control: temephos).

The use of essential oils from aromatic, medicinal, and biocidal plants for vector control provides an effective alternative approach to minimize the adverse environmental and health impacts of chemical pesticides (Nathan, 2007; Sanei-Dehkordi et al., 2016). These essential oils contain secondary metabolites that function as botanical insecticides, and several recent studies have highlighted their potential in combating various disease vectors, such as *C. pipiens* (Cheng et al., 2003; Dias and Moraes, 2014). In this study, the results from the susceptibility test revealed that the essential oils of the three *Artemisia* species exhibited significant larvicidal potential against *C. pipiens*, a major vector of human diseases. The mortality rate of *C. pipiens* larvae increased proportionally with the concentration of essential oils used, as shown in **Figure 1**. For instance, *A. negrei* essential oil demonstrated remarkable efficacy, achieving a 98.33% mortality rate at a concentration of 150 mg/L. This suggests a strong biocidal activity, likely due to the presence of potent bioactive compounds. In comparison, *A. campestris* essential oil exhibited mortality ranging from 18.33% at 100 mg/L to 98.33% at 1000 mg/L, indicating increased efficacy with higher concentrations, although its performance was slightly lower than that of *A. negrei* (Zahran et al., 2017b). Similarly, *A. herba alba* Asso oil showed a mortality rate ranging from 21.67% at 250 mg/L to 100% at 1500 mg/L, an impressive result, although higher concentrations were required for maximum efficacy. These findings confirm that essential oils from *Artemisia* species have significant potential for controlling mosquito larvae, though notable differences exist in the effective concentrations among the species (Sayah et al., 2014).

The LC_50_ values (with 95% confidence intervals) at the larval stage endpoint were 44.562 ± 0.141 mg/L [15.789; 125.763] for *A. negrei*, 326.596 ± 0.308 mg/L [34.222; 3116.82] for *A. campestris*, and 562.554 ± 0.221 mg/L [111.225; 2845.29] for *A. herba-alba* Asso, as shown in the statistical **Table 3**. These values indicate that *A. negrei* is the most toxic of the three oils tested, with an LC_50_ value below 100 ppm, making it a promising candidate for use as a natural insecticide against mosquitoes. Furthermore, the LC_90_ values (with 95% confidence intervals) for these essential oils were 149.181 ± 0.097 mg/L [72.924; 305.18] for *A. negrei*, 1041.58 ± 0.167 mg/L [305.941; 3546.08] for *A. campestris*, and 5202.7 ± 0.12 mg/L [2150.43; 12587.3] for *A. herba-alba* Asso (**Table 3**), confirming that *Artemisia* oils exhibit increasing larvicidal effectiveness with higher concentrations (Traboulsi et al., 2005). The LC_10_ values (with 95% confidence intervals) were also determined, and they were 44.562 ± 0.141 mg/L [15.789; 125.763] for *A. negrei*, 102.407 ± 0.641 mg/L [0.936; 11200.3] for *A. campestris*, and 237.108 ± 0.449 mg/L [8.794; 6392.32] for *A. herba-alba* Asso (**Table 3**). These data reinforce the idea that the effectiveness of the essential oils varies significantly depending on concentration and chemical composition (Michaelakis et al., 2009).

**Table 3:** Lethal concentrations (LC_10_, LC_50_, and LC_90_) of the essential oils of *A. negrei*, *A. herba-alba* Asso, and *A. campestris* after 24 hours of exposure to *C. pipiens* larvae.

Essential oil	Concentrations (mg/L)	LC_10_ (mg/L) CI	LC_50_ (mg/L) CI	LC_90_ (mg/L) CI	Regression line equation	Calculated Chi2 (χ2)
*A. herba-alba* Asso	(250;500;750;1000;1500)	237.108 ± 0.449 [8.794;6392.32]	562.554 ± 0.221 [111.225;2845.29]	1334.70 ± 0.112 [586.524; 3037.24]	3.41595 * X – 9.39434	22.560
*A. campestris*	(100;250; 500;750; 1000)	102.407 ± 0.641 [0.936;11200.3]	326.596 ± 0.308 [34.222;3116.82]	1041.58 ± 0.167 [305.941;3546.08]	2.54471 * X - 6.39744	28.092
*A. negrei*	(25;50;75;100;150)	44.562 ± 0.141 [15.789;125.763]	81.534 ± 0.065 [50.411;131.871]	149.181 ± 0.097 [72.924;305.18]	4.88512 * X - 9.33711	20.880
Temephos (Control+)	(0.0078;0.0,156;0.0312;0.0625;0.125)	0.0086 ± 0.15 [0.0031;0.0144]	0.0256 ± 0.076 [0.0157; 0.0338]	0.0759 ± 0.053 [0.0608; 0.1029]	2.71605 * X + 4.32301	7.183

LC_10_ lethal concentration that kills 10% of exposed larvae. LC_50_ lethal concentration that kills 50% of exposed larvae; LC_90_: lethal concentration that kills 90% of exposed larvae; CI: 95% confidence intervals. The goodness-of-fit of the log-probit model was evaluated using the chi-square (χ²) values automatically calculated by Windl 2.0. These values indicated that the model adequately fit the observed larval mortality data.

The chi-square test showed no significant differences at the 5% level, suggesting that the model used is well suited and that the results are reliable for evaluating the larvicidal effectiveness of the tested essential oils. The lack of significance may also indicate that the variation in mortality is primarily due to the concentration of the essential oil rather than other external factors or experimental biases (Koliopoulos et al., 2010).

The larvicidal activity of the essential oils from the three *Artemisia* species tested revealed differentiated results. *A. negrei* exhibited strong larvicidal effectiveness, with an LC_50_ value of less than 100 mg/L, comparable to that of well-documented bioactive insecticide products (Dias and Moraes, 2014; Chebbac et al., 2024b). This high efficacy can be attributed to the presence of α-pinene, a compound specific to *A. negrei*, which exerts neurotoxic effects on *C. pipiens* larvae (Lucia et al., 2007). The synergy between α-pinene and other compounds, such as 1,8-cineole, enhances the larvicidal efficiency of *A. negrei*, as corroborated by Amal Ramzi’s study (Ramzi et al., 2022). In contrast, *A. herba-alba* Asso contains bioactive compounds such as β-thujone and fenchone, but their high concentrations did not result in the same pronounced larvicidal effect observed with *A. negrei*. Although β-thujone is known for its insecticidal properties (Chebbac et al., 2023), the presence of limonene in *A. herba-alba* Asso seems to enhance the larvicidal efficacy of this essential oil, although to a lesser extent compared to *A. negrei*. Previous studies have reported that the essential oil of *A. herba-alba* Asso, while rich in α-thujone and limonene, contains little or no α-pinene (Paolini et al., 2010). Monoterpenes such as α-pinene have been shown to exert significant larvicidal activity against mosquito larvae, including *C. pipiens* and *Aedes aegypti* (Govindarajan et al., 2016; Alami et al., 2025). Therefore, the relatively low content of α-pinene in *A. herba-alba* Asso may contribute to its lower larvicidal effectiveness compared to *A. negrei*, which contains α-pinene along with camphene and camphor, likely acting synergistically to enhance larvicidal potential (Ramzi et al., 2022). The high concentration of β-thujone may play a role in the relative effectiveness of *A. herba-alba* Asso, but the synergy between α-pinene and other compounds in *A. negrei* appears to be a key factor in achieving greater efficacy. *A. campestris*, on the other hand, exhibited more moderate larvicidal activity. The absence of limonene and α-pinene in this species is likely a limiting factor. Although it contains compounds such as camphene and 1,8-cineole, their concentrations and synergistic interactions with other bioactive molecules in *A. campestris* were insufficient to produce the same powerful larvicidal effect observed with *A. negrei*. In summary, the larvicidal efficacy of *A. negrei* can be attributed to the synergy between specific compounds such as α-pinene, camphene, and camphor, which maximize its insecticidal potential. In contrast, *A. herba-alba* Asso and *A. campestris* exhibit less pronounced larvicidal activity, likely due to the absence of α-pinene and differences in the concentrations of other monoterpenes. The observed differences in larvicidal activity among the essential oils can be largely attributed to the presence and relative abundance of compounds previously reported to exhibit larval activity. It should be noted that some confidence intervals, particularly for LC10 values of *A. campestris* and *A. herba-alba* Asso, are relatively wide. This likely reflects natural variability in larval sensitivity and the limited number of larvae used per test, which can amplify variation at lower concentrations. Acknowledging this variability provides transparency regarding the experimental data without affecting the overall conclusions about the relative larvicidal efficacy of the essential oils (Coelho et al., 2025).

In particular, the presence of *α*-pinene, camphor, and *α*-thujone appears to play a key role in enhancing the larvicidal potential of *A. negrei*, likely through synergistic interactions. While testing the major components individually could provide additional insights into their specific contributions, the overall activity of the complete essential oil reflects the combined effects of all bioactive constituents.

As a positive control, Temphos, the most commonly used insecticide locally in Morocco, exhibited very high insecticidal activity against *C. pipiens* larvae, with LC50, LC90, and LC10 values of 0.0256 ± 0.076 [0.0157; 0.0338] mg/L, 0.0759 ± 0.053 [0.0608; 0.1029] mg/L, and 0.0086 ± 0.15 [0.0031; 0.0144] mg/L, respectively (**Table 2**). However, the widespread development of resistance to Temphos and other conventional insecticides, such as Malathion, Fenitrothion, and Fenthion (Lee, 2006), limits their effectiveness. Despite lower potency compared to Temphos, the essential oils demonstrated promising larvicidal activity, supporting their potential as environmentally friendly alternatives for mosquito control, particularly in light of concerns over chemical insecticide resistance, environmental pollution, and human health risks (Kordali et al., 2006; Chebbac et al., 2023).

### 3.4 Molecular docking studies

To gain deeper insight into the insecticidal potential of the major constituents identified in the *Artemisia* species, molecular docking simulations were carried out to assess their binding affinities and elucidate the key interactions involved in the inhibition of gamma-aminobutyric acid transaminase (GABA-T). This mitochondrial enzyme plays a pivotal role in the catabolism of gamma-aminobutyric acid (GABA), the primary inhibitory neurotransmitter in the insect central nervous system. The choice of GABA-T as a molecular target was motivated by its well-established involvement in insect neurophysiology and its frequent implication in the mode of action of neurotoxic insecticides and plant-derived bioactive compounds. Inhibiting GABA-T leads to the accumulation of GABA, thereby disrupting neuronal signaling, reducing neural excitability, impairing motor function, and ultimately leading to the death of the larvae (Taktak et al., 2022). **Table 4** presents the docking results for the major compounds identified in the three *Artemisia* species, as well as the standard insecticide temephos within the active site of the GABA-T enzyme. The binding affinity values ranged from -4.5 to -6.8 kcal/mol, reflecting a generally strong interaction profile across the tested compounds. Notably, two constituents from *A. negrei*, namely α-copaene and curcuphenol, exhibited the most favorable binding energies (–6.8 kcal/mol), surpassing that of temephos (–6.5 kcal/mol). These results align with experimental data, showing that *A. negrei* exhibits significantly higher insecticidal activity compared to *A. campestris* and *A. herba-alba* Asso. The two compounds with the best docking scores can be considered the primary contributors to the enhanced GABA-T inhibitory activity observed in *A. negrei*, explaining its superior efficacy relative to the other species.

**Table 4:** Binding affinities of the major compounds identified in the studied *Artemisia* species and temephos within the active site of GABA-T enzyme (PDB ID: 1SF2).

Artemisia species	Compound	Docking score kcal/mol
*A. negrei*	β-Thujone	-6.0
	α-Thujone	-5.9
	Camphor	-5.6
	Octacosane	-4.5
	1,8 Cineole	-6.1
	α-Pinene	-6.0
	α-Copaene	-6.8
	Camphene	-6.3
	Limonene	-6.1
	Fenchone	-6.4
	Limonen-4-ol	-6.1
	Curcuphenol	-6.8
*A. campestris*	Camphor	-5.6
	1,8 cineole	-6.1
	β-Thujone	-6.0
	borneol	-5.7
	isophoron	-6.1
	menth-en-1-ol	-6.1
	camphene	-6.3
*A. herba-alba* Asso	α-Thujone	-5.9
	terpinene-4-ol	-6.0
	terpineol	-6.3
	p-cymen-7-ol	-6.3
	Camphor	-5.6
	camphene	-6.3
	Limonene	-6.1
	Fenchone	-6.4
Standard insecticide	Temephos	-6.5

**Figure 2:**
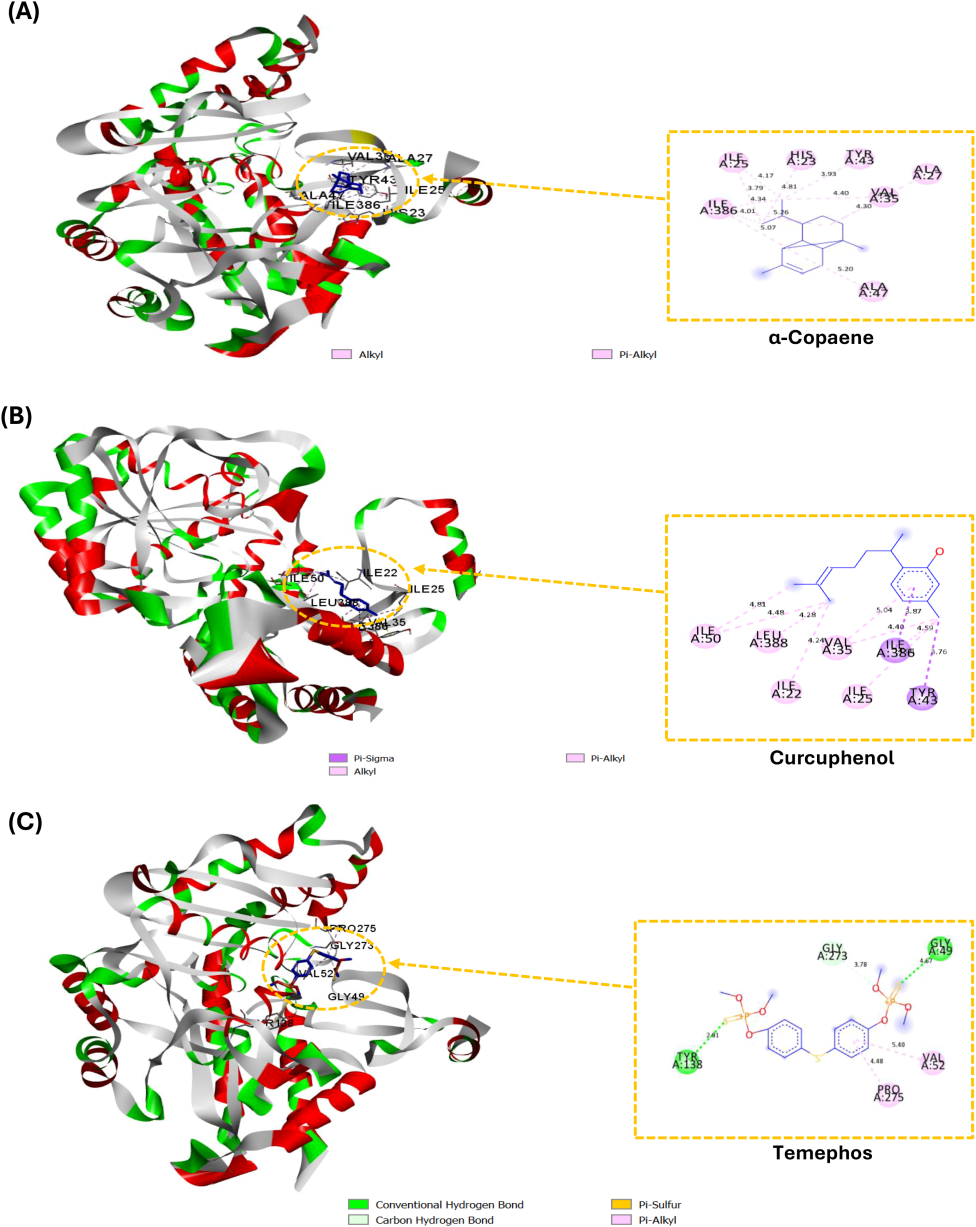
Docking views of α-Copaene **(A)**, Curcuphenol **(B)**, and Temephos **(C)** on the binding site of GABA-T (PDB ID: 1SF2). Right are the 2D interaction diagrams, and left are the complex structures in 3D.

To better elucidate the molecular basis underlying the superior insecticidal efficacy of *A. negrei*, we performed a comparative analysis of the binding interactions established by its two most active constituent’s α-copaene and curcuphenol with those formed by temephos within the active site of GABA-T. As shown in **Figure 2**, α-copaene (A) is predominantly stabilized within the binding pocket through hydrophobic interactions, including multiple alkyl and π-alkyl contacts involving key non-polar residues such as Val434 (4.28 Å), Ala437 (3.79–5.20 Å), Ile386 (3.75–4.01 Å), Leu223 (5.08 Å), and Ala428 (3.94 Å). These close contacts suggest that α-copaene fits snugly within a hydrophobic cleft of the enzyme. Curcuphenol (B) also displays a robust hydrophobic interaction profile, characterized by π-alkyl and π-sigma interactions with residues such as Ile22 (4.91 Å), Leu25 (4.98 Å), Leu50 (5.96 Å), Val58 (4.39–4.90 Å), and Ala385 (4.74–5.06 Å). The spatial arrangement of these residues around the aromatic system of curcuphenol likely contributes to ligand stabilization through van der Waals forces and π-stacking. Regarding temephos (C), it adopts a different binding mode, primarily stabilized through polar interactions. These include conventional hydrogen bonds with Gly149 (3.76 Å) and carbon hydrogen bonds with Lys273 (3.17 Å), Gly273 (3.42 Å), and Lys152 (3.54 Å). Additionally, a π-sulfur interaction is observed with Cys148 (5.68 Å). These interactions support ligand anchoring within the active site.

### 3.5 Molecular dynamics simulations

The two top-performing ligand–GABA-T complexes, selected based on their binding affinities, along with the standard insecticide, were subjected to 100 ns molecular dynamics simulations to assess their conformational stability and interaction profiles. The resulting trajectories were analyzed using key parameters such as the root mean square deviation (RMSD) and root mean square fluctuation (RMSF), which provide insight into the overall structural stability and local flexibility of the complexes (Guerguer et al., 2025b). In addition, enzyme–ligand interaction analyses throughout the simulation period enabled the characterization of the persistence of crucial molecular contacts essential for inhibitory activity (Bouribab et al., 2024). Together, these parameters offer a comprehensive view of the dynamic behavior of each complex and support the evaluation of these compounds as potentially stable and effective GABA-T inhibitors.

### 3.6 Root-mean square deviation

**Figure 3:**
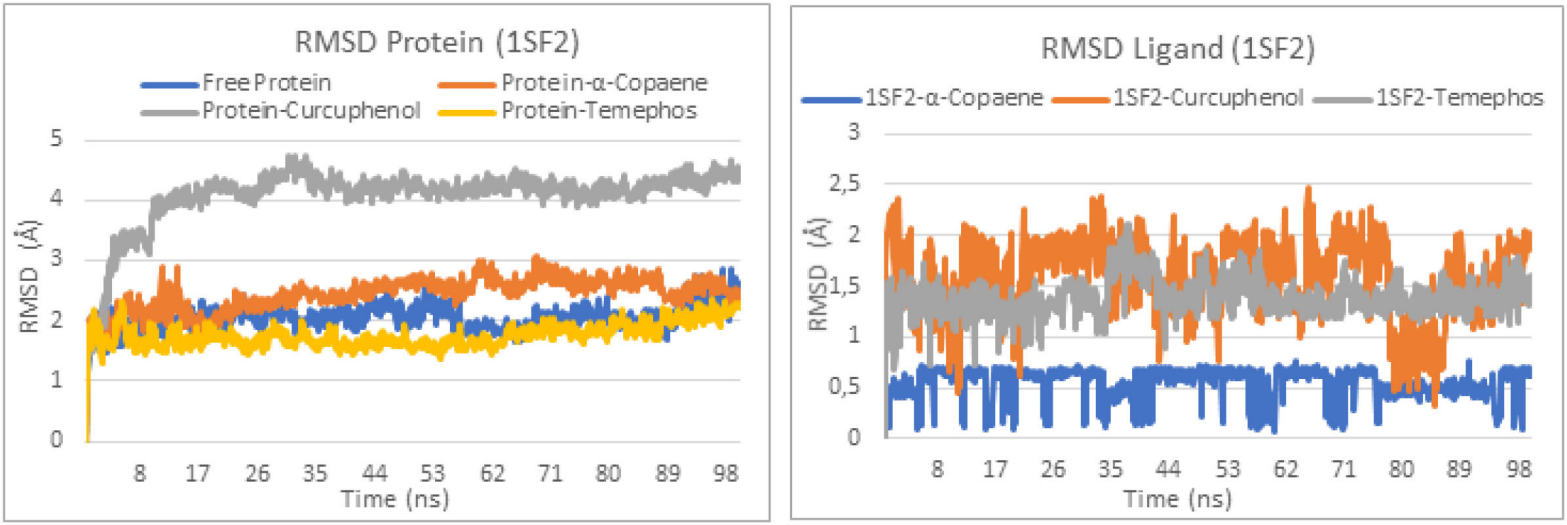
The RMSD protein and RMSD ligand plots of complexes 1SF2-α-Copaene, 1SF2-Curcuphenol, and 1SF2-Temephos.

The Root Mean Square Deviation (RMSD) is a key parameter used to evaluate the structural stability of ligand–protein complexes during molecular dynamics simulations. Compared to the free protein, which maintains a relatively stable RMSD around 2.7 Å, the complex formed with α-copaene exhibits remarkable structural stability, with a slightly lower average RMSD of approximately 2.5 Å. This performance is comparable to, or even slightly better than, that of the temephos complex which also remains stable around 2.7 Å, indicating a similarly well-preserved structure. In contrast, the curcuphenol complex displays significantly higher fluctuations, reaching up to 4.5 Å, suggesting notable instability. These findings are further supported by the ligand RMSD analysis: α-copaene remains tightly bound within the active site, maintaining an RMSD below 1 Å throughout the simulation, reflecting exceptional positional stability. Temephos exhibits intermediate stability, with a mean RMSD around 1.5 Å, while curcuphenol shows greater variation, with values ranging between 1.5 and 2.5 Å, indicative of a certain instability in its interaction within the active site (**Figure 3**).

### 3.7 Root-mean square fluctuation

**Figure 4:**
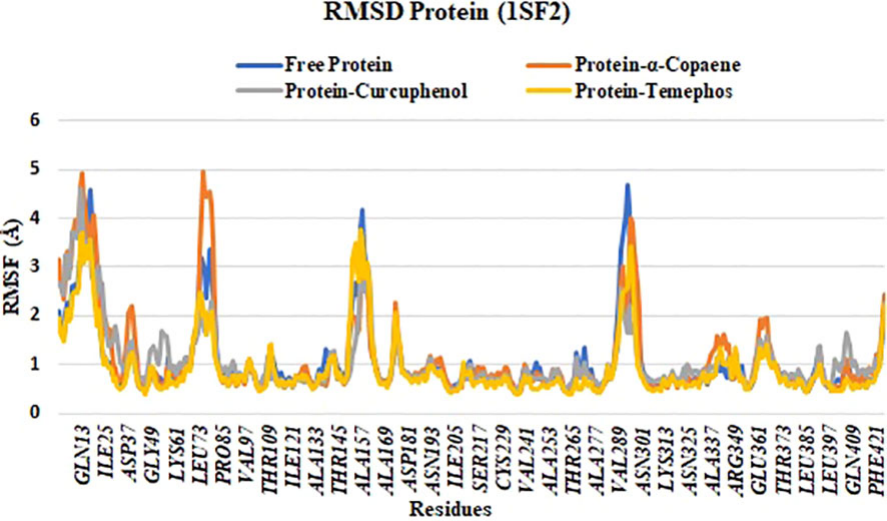
The RMSF protein plot of complexes 1SF2-α-Copaene, 1SF2-Curcuphenol, and 1SF2-Temephos.

The analysis of root-mean-square fluctuation (RMSF) revealed that all studied ligands induced flexibility in similar regions of the protein. Fluctuations ranging from 2 to 5 Å were predominantly observed in regions corresponding approximately to residues Ile22, Tyr43, Pro85, Leu148, and Gly295. α-copaene appeared to induce a localized increase in flexibility. In contrast, temephos maintained a fluctuation profile closely resembling that of the unbound free protein. Conversely, curcuphenol exhibited a slightly less consistent pattern, with reduced fluctuations compared to the other ligands (**Figure 4**).

### 3.8 Protein-ligand interactions

**Figure 5:**
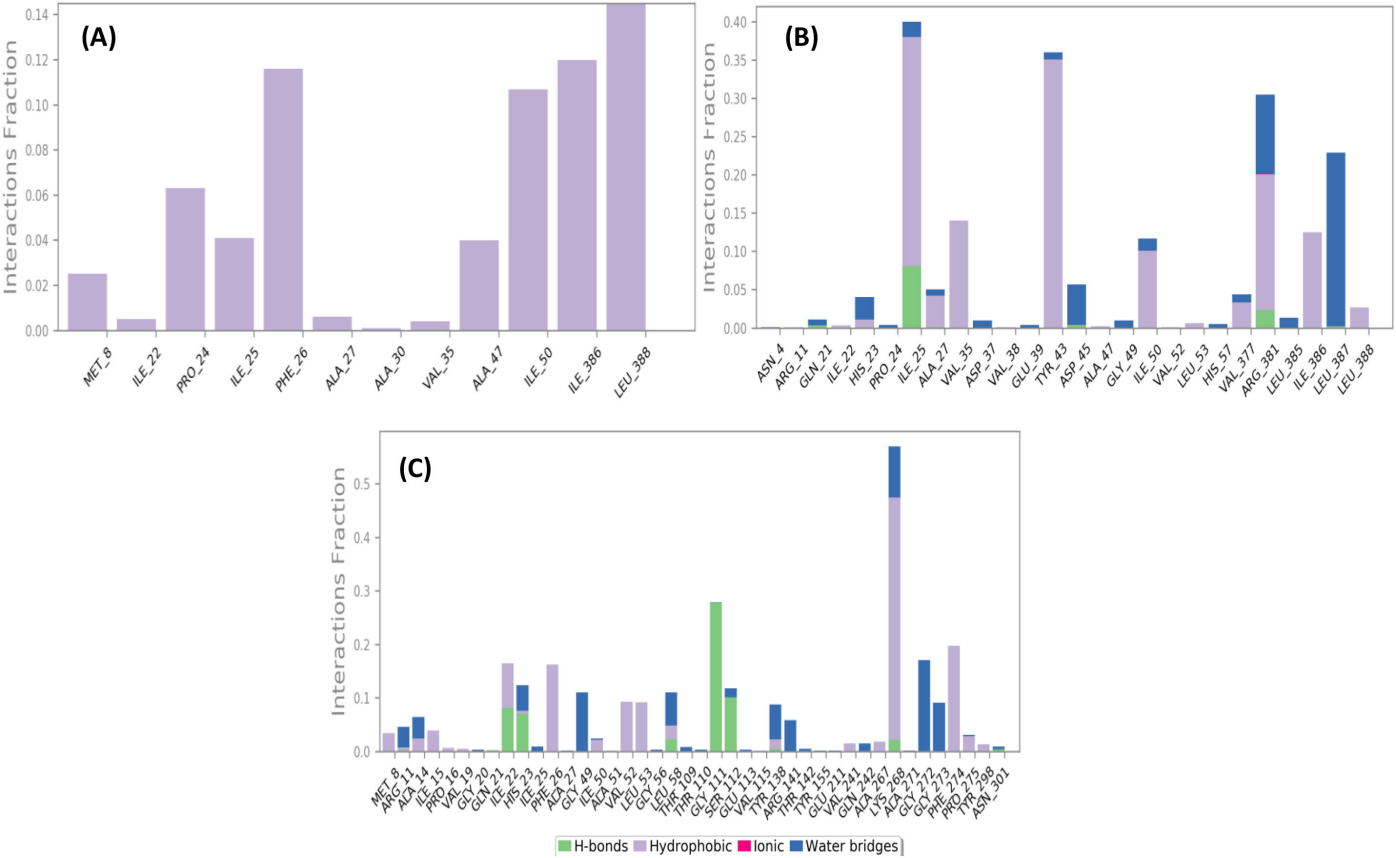
Protein–ligand interactions of complexes 1SF2-α-Copaene **(A)**, 1SF2-Curcuphenol **(B)**, and 1SF2-Temephos **(C)**.

The analysis of protein–ligand interactions enabled the identification of key residues involved in the stabilization of each complex. In the case of the α-copaene complex (**Figure 5A**), only hydrophobic interactions were observed, with notable contributions from residues Leu388, Ile386, and Phe26, exhibiting high interaction fractions of approximately 0.14, 0.12, and 0.11, respectively. In comparison, the complex formed with curcuphenol (**Figure 5B**) displays a slightly less diversified interaction profile but remains characterized by prominent hydrophobic contacts involving Tyr43 (≈ 0.31) and Pro24, along with the presence of several water bridges, particularly around residues Ile386 and Leu387. As for the complex with temephos (**Figure 5C**), it exhibits a balanced interaction profile, combining hydrogen bonds with Ile22 (≈ 0.13), Gly111 (≈ 0.52), and Ser112, with strong hydrophobic interactions involving Phe274 and Lys268. Additionally, the abundance of water bridges particularly those formed with Gly272 and Gly274. As a result, the diversity and complementarity of interactions observed in each system likely account for the favorable dynamic behavior and overall stability maintained throughout the simulation.

## 4 Conclusion

This study demonstrates that essential oils from the three endemic Moroccan *Artemisia* species exhibit significant larvicidal potential against *C. pipiens*, with *A. negrei* showing the highest efficacy. The observed bioactivity appears closely linked to the presence of specific mono- and sesquiterpenes, suggesting that the chemical composition plays a critical role in larvicidal effectiveness. These findings highlight the potential of *Artemisia* essential oils as natural, environmentally friendly alternatives to conventional chemical insecticides.

Overall, this research highlights the potential of essential oils from species of the genus *Artemisia* as promising bioinsecticides against mosquitoes. These results suggest a natural alternative that could contribute to sustainable control strategies and integrated vector management programs, subject to further studies aimed at optimizing yields, formulations and larger-scale evaluation.

## Data Availability

The original contributions presented in the study are included in the article/supplementary material. Further inquiries can be directed to the corresponding authors.
